# Treatment of Malarious Fevers by Subcutaneous Injection of Solution of Quinia

**Published:** 1865-02

**Authors:** 


Treatment of Malarious Fevers by Subcutaneous Injection of Solution of Quinia.—Mr. Moore, of the Bombay Service, states (*The Indian Annals of Medical Science*, April, 1864) that he has been latterly using quinia hypodermetically for the cure of malarious fevers with success.

"I use," he says, "the strongest solution of quinine which can be prepared, viz.: thirty grains of quinine, ten or twelve drops of sulphuric acid, and half an ounce of water. Of this, previously strained, I inject from half a drachm to a drachm, the former quantity containing somewhat less than four grains of the active agent. With the exception of a little sulphate of soda, if the bowels are confined, I have used no other remedies, in complicated cases of any type of malarious fever. When the spleen is enlarged, or a leucocythemic condition manifest, I prescribe, as an additional curative agent, one or other of the preparations of iron."

